# Integrating broad‐scale data to assess demographic and climatic contributions to population change in a declining songbird

**DOI:** 10.1002/ece3.5975

**Published:** 2020-02-11

**Authors:** James F. Saracco, Madeleine Rubenstein

**Affiliations:** ^1^ The Institute for Bird Populations Point Reyes Station CA USA; ^2^ USGS National Climate Change & Wildlife Science Center Reston VA USA

**Keywords:** Avian demography, *Cardellina pusilla*, climate variation, integrated population model, Monitoring Avian Productivity and Survivorship, North American Breeding Bird Survey, transient life table response experiment

## Abstract

Climate variation and trends affect species distribution and abundance across large spatial extents. However, most studies that predict species response to climate are implemented at small spatial scales or are based on occurrence‐environment relationships that lack mechanistic detail. Here, we develop an integrated population model (IPM) for multi‐site count and capture‐recapture data for a declining migratory songbird, Wilson's warbler (*Cardellina pusilla*), in three genetically distinct breeding populations in western North America. We include climate covariates of vital rates, including spring temperatures on the breeding grounds, drought on the wintering range in northwest Mexico, and wind conditions during spring migration. Spring temperatures were positively related to productivity in Sierra Nevada and Pacific Northwest genetic groups, and annual changes in productivity were important predictors of changes in growth rate in these populations. Drought condition on the wintering grounds was a strong predictor of adult survival for coastal California and Sierra Nevada populations; however, adult survival played a relatively minor role in explaining annual variation in population change. A latent parameter representing a mixture of first‐year survival and immigration was the largest contributor to variation in population change; however, this parameter was estimated imprecisely, and its importance likely reflects, in part, differences in spatio‐temporal distribution of samples between count and capture‐recapture data sets. Our modeling approach represents a novel and flexible framework for linking broad‐scale multi‐site monitoring data sets. Our results highlight both the potential of the approach for extension to additional species and systems, as well as needs for additional data and/or model development.

## Graphical abstract

We develop an integrated population model for age‐specific capture and capture‐recapture data from the Monitoring Avian Productivity and Survivorship (MAPS) program, count data from the North American Breeding Bird Survey (BBS), and remote‐sensed and modeled climate data. We apply the model to data from a declining bird species, Wilson's Warbler (*Cardellina pusilla*) in three distinct genetic populations in western North America. Results highlight the potential of the approach for extension to additional species and systems, as well as a need for additional data and/or model development.

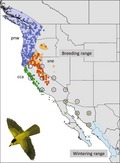

## 1 INTRODUCTION

Widespread population declines, range retractions, and extinctions highlight an urgent need to better understand drivers of wildlife population dynamics (Ceballos, Ehrlich, & Dirzo, 2017; Tittensor et al., 2014). Climate variation and trends can play a crucial role in determining population trajectories (Stephens et al., 2016). Most studies that relate climate to populations across large spatial extents are based on occurrence or count data (Dawson, Jackson, House, Prentice, & Mace, 2011; Pacifici et al., 2015). These data types have become relatively common and available across large spatial extents (Sullivan et al., 2009) and can be used to model both population state and demographic rate parameters (Dail & Madsen, 2011; Royle, 2004). However, estimates of demographic rates from such models may not always be reliable (Barker, Schofield, Link, & Sauer, 2018; Dennis, Morgan, & Ridout, 2015; Zipkin et al., 2014). Capture‐mark‐recapture (CMR) data provide additional information for modeling demographic rates, providing a more mechanistic link between population dynamics and climate than models based on occurrence or count data alone (Amburgey et al., 2018; Buckley et al., 2010; McMahon et al., 2011; Selwood, McGeoch, & Mac Nally, 2015). However, CMR data are less available and are relatively costly to obtain across large spatial extents. Analyses that incorporate strengths of different data types and models have the potential to improve the inferences about population dynamics.

Integrated population models (IPMs) provide a formal framework for jointly modeling independent count and CMR data (Besbeas, Freeman, Morgan, & Catchpole, 2002; Hostetler, Sillett, & Marra, 2015; Schaub & Abadi, 2011) along with climate predictors of demographic rates (Zipkin & Saunders, 2018). With one recent exception (Zhao, Boomer, & Royle, 2019), IPMs that have included climate covariates of demographic rates have been limited to population studies across relatively small spatial extents (Woodworth, Wheelwright, Newman, Schaub, & Norris, 2017) or have modeled multiple local populations independently (Weegman, Arnold, Dawson, Winkler, & Clark, 2017). Data from long‐running national and continental scale avian monitoring programs (Dunn et al., 2005; Gregory et al., 2005; Robinson, Julliard, & Saracco, 2009; Sauer & Link, 2011) present a unique opportunity for extending IPMs to broad‐scale applications. However, development and application of IPMs for broad‐scale bird‐monitoring data are challenging because of a variety of sampling issues, including mismatches in sizes and spatio‐temporal distribution of sampling areas between monitoring data sets and properly accounting for observation error associated with multi‐site, multi‐observer studies (Ahrestani, Saracco, Sauer, Royle, & Pardieck, 2017; Robinson, Morrison, & Baillie, 2014). Additionally, IPMs developed for local scale studies based on binomial and Poisson population processes may not be appropriate at regional scales where population responses represent averages of local studies (Zhao et al., 2019).

Analyses of broad‐scale IPMs require spatial stratification at ecologically relevant scales. Stratification decisions may involve geopolitical boundaries that are meaningful in conservation applications (e.g., state × bird conservation region; Sauer & Link, 2011). One approach would be to stratify on as fine of a resolution as possible and model spatial structure explicitly (Bled, Sauer, Pardieck, Doherty, & Royle, 2013; Saracco, Royle, DeSante, & Gardner, 2010). This approach offers the advantage of allowing poststratification summaries across any larger spatial resolution of interest but can be computationally expensive. Alternatively, stratification may be based on natural spatial structuring of population dynamics (Rushing, Ryder, Scarpignato, Saracco, & Marra, 2016) or genetics (Ruegg, Harrigan, Saracco, Smith, & Taylor, unpublished data; Ruegg et al., 2014). Spatial structuring of migratory species may be maintained (strong migratory connectivity) or dissolved (weak connectivity) between breeding and nonbreeding seasons. Effectively linking environmental covariates to demography in these species requires understanding of spatial structuring throughout the annual cycle (Cohen et al., 2018; Hostetler et al., 2015; Ruegg et al., 2014) and consideration of potential “carry‐over effects,” whereby environmental conditions experienced in one season affect demographic rates and population changes in a subsequent season (Norris & Marra, 2007).

**Figure 1:**
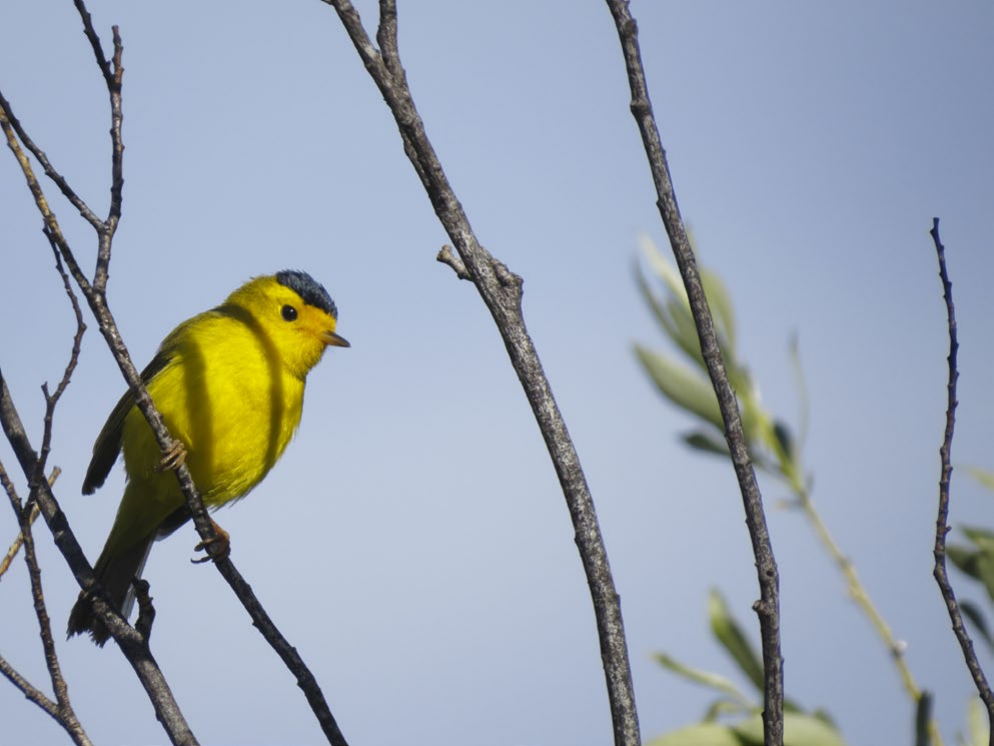
Adult male Wilson's warbler in California's Sierra Nevada. Photography credit: Gabriel Gonzalez

Here, we develop an IPM for data from the North American Breeding Bird Survey (BBS; Pardieck, Ziolkowski, Hudson, & Campbell, 2016) and the Monitoring Avian Productivity and Survivorship program (MAPS; DeSante & Kaschube, 2009) to assess potential climate impacts on demographic rates and population dynamics of a migratory songbird species, Wilson's warbler (*Cardellina pusilla*), within three distinct genetic regions of the western United States (Ruegg et al., 2014). Wilson's warbler (Figure 1) is a good candidate for development of a climate‐informed IPM, as it is well‐represented in BBS and MAPS data sets in the western United States; its patterns of migratory connectivity are relatively well understood (Ruegg et al., 2014); its population has declined in recent decades (Sauer et al., 2017); and it has been designated as an “at‐risk” species due to climate change (http://climate.audubon.org/birds). We included remote sensed and modeled climate covariates in models of demographic rates. We hypothesized that breeding productivity would depend on drought conditions on the wintering grounds (a carry‐over effect) and on spring temperature (Saracco, Siegel, Helton, Stock, & DeSante, 2019; Socolar, Epanchin, Beissinger, & Tingley, 2017) and that adult survival would depend on winter drought and wind conditions during spring migration (Drake, Rock, Quinlan, Martin, & Green, 2014; Huang, Bishop, McKibbin, Drake, & Green, 2017; LaManna, George, Saracco, Nott, & DeSante, 2012). We used transient life table response experiments (LTREs) to decompose variation in population growth rates among vital rate and demographic structure components and to examine how these demographic contributions depended on climate covariates (Koons, Arnold, & Schaub, 2017; Koons, Iles, Schaub, & Caswell, 2016).

## 2 METHODS

### 2.1 Bird‐monitoring data and focal species

**Figure 2:**
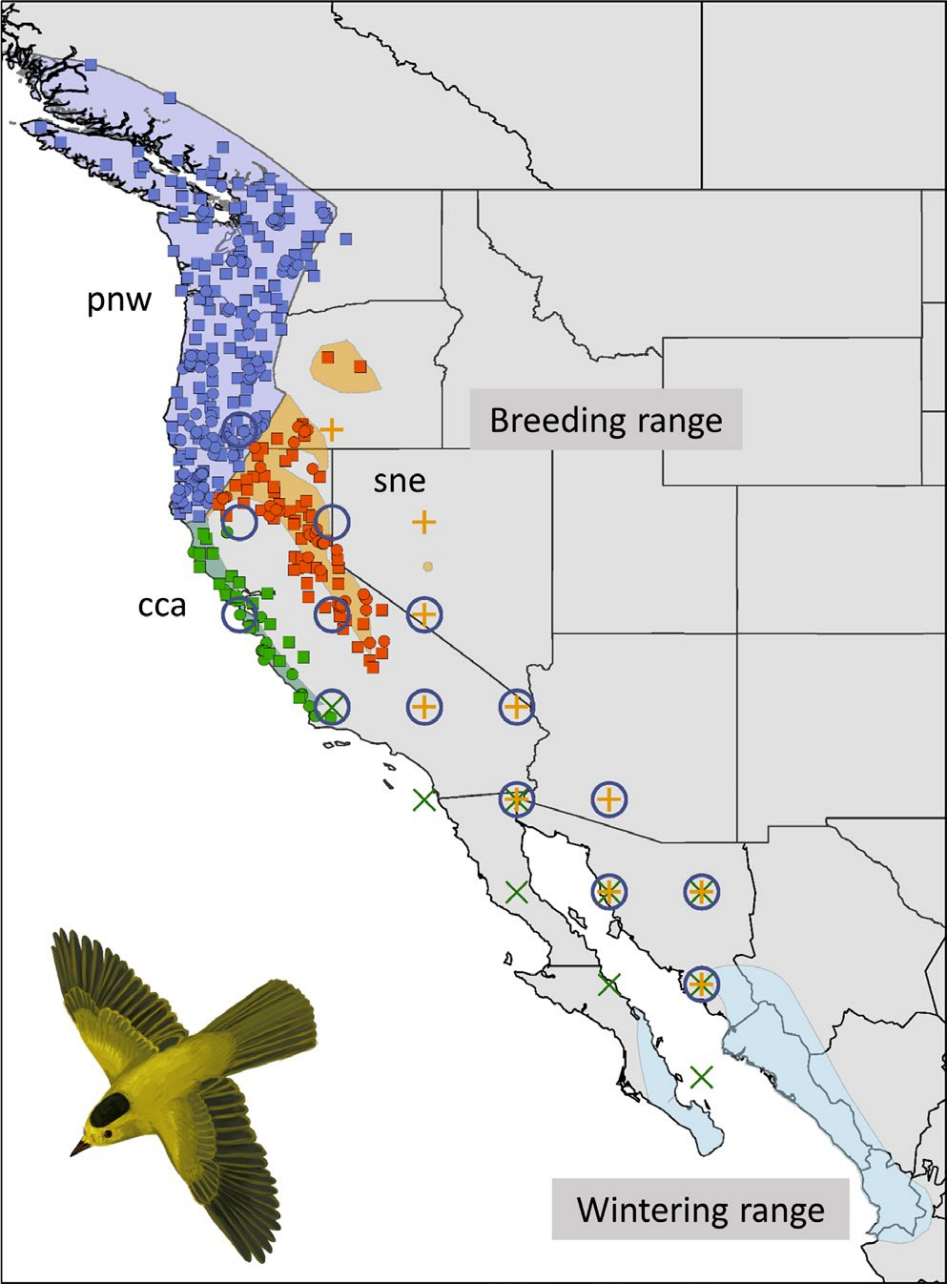
Breeding Bird Survey (BBS) routes (squares) and MAPS stations (circles) sampled between 1992 and 2008 where Wilson's warbler was detected or captured. The three genetically distinct breeding regions (Ruegg et al., 2014) included the Pacific Northwest (pnw; purple), coastal California (cca; blue), and the Sierra Nevada (sne; orange). Birds of all three breeding regions winter in northwest Mexico (blue), although migratory connectivity data suggest that only the cca breeding region includes the southern Baja California portion of the wintering range. Points for which spring migration wind data were used are shown for each breeding region (purple circles for pne; green ×'s for cca; orange +'s for sne)

Our analysis incorporates annual counts of adult birds from the BBS and capture‐recapture data on adult birds and age‐specific capture data from the MAPS program. The BBS is a roadside bird survey established in 1966 that provides data on the status and population trends of >420 bird species (Sauer & Link, 2011; Sauer et al., 2017); it is a core component of North American bird conservation efforts (Rosenberg et al., 2016). The MAPS program, established in 1989 and standardized in 1992, uses data from a cooperative network of mist‐netting and bird‐banding stations to provide information on demographic rates of >100 landbird species (DeSante & Kaschube, 2009). Here, we analyze MAPS and BBS data for Wilson's warbler stratified by three genetically distinct breeding regions that overwinter in northwestern Mexico (Ruegg et al., 2014; Figure 2; Table 1). Although MAPS and BBS data were available from additional genetic breeding regions, we limited our analysis to just these three regions because other breeding populations regularly utilize wintering ranges farther south than our climate covariate data set extended (Wang, Hamann, Spittlehouse, & Carroll, 2016; Wang, Hamann, Spittlehouse, & Murdock, 2012). For illustration of our model, we limit the time window of our analysis to the 17 years 1992–2008 based on the earliest year and latest years of vetted MAPS data available when the analysis was undertaken (updated verified MAPS data base expected in 2020).

**Table 1:** Summary of the BBS and MAPS data sets used in the integrated population model

Region	BBS data		MAPS age‐specific capture data			MAPS adult CMR data		
	No. routes	Birds/route	No. stations	No. juvs.	No. adults	No. stations	No. inds.	No. recaps.
Pacific Northwest (pnw)	148	8.07	105	1,919	5,152	66	3,781	483
Sierra Nevada (sne)	58	0.89	43	982	3,826	26	2,595	408
Coastal California (cca)	21	13.15	29	2,424	2,449	15	4,424	401

Note

The MAPS adult CMR data set was restricted to stations operated ≥4 years.

### 2.2 Climate data

We calculated overwintering and breeding season climate covariates from the ClimateNA database (https://sites.ualberta.ca/~ahamann/data/climatena.html). ClimateNA uses bilinear interpolation of monthly gridded climate data (Daly et al., 2008; Hutchinson, 1989) and local elevation adjustments to provide climate metrics for individual points (Wang et al., 2016, 2012). An advantage of using this data set is the availability of interpolated values for fine spatial resolution and projected climate estimates for future time periods to assess population viability under climate change scenarios.

#### 2.2.1 Overwintering season

Drought associated with the dry season may limit vital rates of birds overwintering in western Mexico (LaManna et al., 2012; Nott, Desante, Siegel, & Pyle, 2002); and winter drought conditions in this region are expected to become more severe in the coming decades (IPCC, 2014). To characterize annual winter drought conditions, we used Hargreave's Climate Moisture Deficit (cmd), a derived variable calculated as the monthly summed difference between atmospheric evaporative demand (Hargreaves & Samani, 1982) and precipitation. We extracted winter (December–February) cmd values for 10,000 random points across the winter range for 1992–2008 and for “normal” (i.e., mean) values for 1961–1990. We then calculated the deviation of the annual value from the normal value to derive a cmd anomaly (cmd) for inclusion in the analysis. For birds that breed in the coastal California region (cca), we used cmd values from both the Baja California and western Mexico portions of the overwintering regions, based on evidence that birds from this breeding region use both of these areas nearly equally during the nonbreeding season (Ruegg et al., unpublished data). For the remaining two breeding regions, we used only cmd values from western Mexico, as birds from these two regions do not appear to use (or use minimally) the Baja California portion of the overwintering range.

#### 2.2.2 Breeding season

We calculated mean spring (February–May) temperature (temp) within each of the breeding regions from averaged ClimateNA values from 1,000 random points sampled within each breeding region. Temperature covariates were among the top explanatory variables in models of projected changes in the distribution of Wilson's warbler under climate change (C. Wilsey and N. Michel, pers. comm.), and spring temperature in particular may affect snowmelt, green‐up, and food availability during the nesting season, which can affect timing of breeding and productivity (Saracco et al., 2019). As with the drought covariate, we subtracted the annual spring temperature values from 1961 to 1990 normal values to derive the covariate used in the analysis described below.

#### 2.2.3 Spring migration

Spring wind conditions may be an important factor affecting survival rates of western Neotropical migrant songbirds (Drake et al., 2014; Huang et al., 2017). We calculated a covariate representing average tailwind conditions experienced during spring migration for each overwintering‐breeding region connection. We used the RNCEP package in R to extract and process 2.5‐degree grid U‐ and V‐wind vector data from the National Centers for Environmental Prediction (NCEP)/National Center for Atmospheric Research (NCAR) Reanalysis data set (Kalnay et al., 1996). We averaged 6‐hr wind vector data, excluding 12:00 p.m. values (when migrating songbirds are normally sedentary) at two atmospheric levels representative of altitudes typically used by migrating songbirds (800 and 950 mb). For grid points linking each breeding‐wintering region (Figure 2), we calculated an average tailwind covariate (tw) for the spring migration period (7 Mar–21 April for cca; 1 April–31 May for sne and pnw) using the NCEP.Tailwind function in RNCEP based on bearings representing centroids of breeding and winter ranges (Kemp et al., 2012).

### 2.3 Model development and implementation

**Figure 3:**
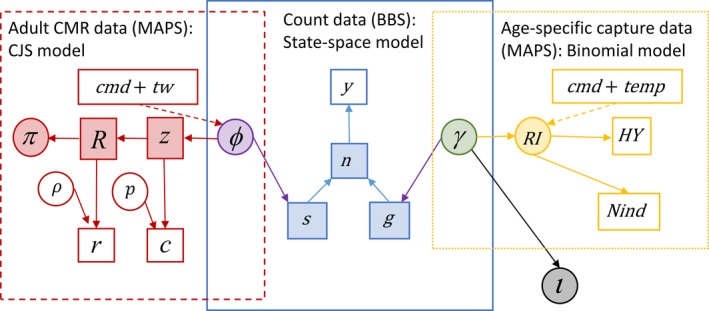
Graphical representation of the hierarchical model that integrates North American Breeding Bird Survey (BBS) and Monitoring Avian Productivity and Survivorship (MAPS) data and includes climate covariates of vital rates. The overall model can be characterized by three sub‐models: (1) a state‐space model for the BBS count data (solid blue); (2) a state‐space Cormack‐Jolly‐Seber (SS CJS) model for MAPS capture‐recapture data of adult birds (left; dashed red); and (3) a binomial model of productivity based on age‐specific MAPS capture data (right; dotted yellow). Additional parameters and hyperparameters accounting for spatial and temporal variation in model components are not shown. Data inputs are represented by open rectangles (*y* = BBS counts; *r* = MAPS observed residency; *c* = MAPS adult capture histories; HY = number of young [hatching year] captures; Nind = number of captures; cmd = winter drought index; tw = tailwind; temp = spring temperature). State variables are represented by shaded rectangles (*R* = residency state; *z* = alive state; *s* = number of survivors; *g* = number of recruits; *n* = *s* + *g*). Parameters associated with observation processes of state‐space models (i.e., “nuisance” parameters) are represented by open circles (*p* = recapture probability; *ρ* = observed residency probability. Estimated population parameters are represented by shaded circles. Residency probability, *π*, and the productivity index, RI, are shaded to match sub‐models informing them. Adult survival probability, *ϕ* and recruitment, γ are shaded intermediate colors to highlight their dependence on information shared between sub‐models. Stochastic relationships (i.e., model likelihoods) are represented by solid arrows. Climate covariate (cmd, tw, and temp) relationships are shown as dashed arrows. First‐year survival/immigration, *ι*, is a latent parameter (black/gray) not directly informed by the monitoring data. See Section 2.3 for detail

Our overall model was comprised of three basic components (Figure 2): (a) A state‐space model for the observed BBS counts and population dynamics; (b) a state‐space transient Cormack‐Jolly‐Seber model applied to MAPS capture histories for adult birds to inform adult survival probabilities; and (c) a model for the recruitment process, partially informed by a binomial model applied to age‐structured MAPS capture data (Figure 3). Data and code for implementing the model and reproducing results are available at https://doi.org/10.21429/04ma-p963.

#### 2.3.1 Count model for BBS data

At the core of the IPM is a state‐space model for the count data (solid blue box in Figure 3). We modeled the counts, *y*, from *i* in 1, …, Nro BBS route × observer combinations; *m* in 1, …, *M* = 3 strata (where strata represent the smallest common scale of inference for the two data sets); and *t* in 1992, …, *T* = 2008 years according to an overdispersed Poisson distribution: $y_{i,m,t}∼\text{Pois}\left(\lambda_{y(i,m,t)}\right)$. We modeled count means as a function of an annual stratum‐level population size index $n_{m,t}$, and effects accounting for observer/route and overdispersion effects (Ahrestani et al., 2017):

$$\log\left(\lambda_{i,m,t}\right)=\log\left(n_{m,t}\right)+\omega_{i}+\eta \left(i,t\right)+\varepsilon_{i,m,t}$$

Following Link and Sauer (2002), the *ω_i_* represents a random observer × route effect, with precision hyperparameter *τ_ω_* (where $\tau =1/\sigma_{\omega }^{2}$); *η* represents a fixed novice observer effect (start‐up‐year effect) multiplied by an indicator variable *I*(*i,t*), where *I*(*i,t*) = 1 for the first year an observer completes a survey on a route and *I*(*i,t*) = 0 on other years; and $\varepsilon_{i,m,t}$ is an error term allowing for extra binomial variation (overdispersion) with precision hyperparameter *τ_ε_*.

We modeled *n_m,t_* at each time step as the sum of surviving adults from the previous year, *s_m,t_* and the number of recruits (local recruits + immigrants) entering the population between years, *g_m,t_*. For the initial time step (*t* = 1992), numbers of survivors and recruits were determined mainly by the count data and (weakly informative; see below) prior distributions. We modeled *s_m,t_* and *g_m,t_* at subsequent time steps based on Gaussian approximations of binomial and Poisson distributions, respectively (Zhao et al., 2019):

$$\begin{array}{c} s_{m,t}{∼\text{Norm}|}_{0}^{+\infty }\left(n_{m,t-1}\times \phi_{m,t-1},n_{m,t-1}\times \phi_{m,t-1}\times \left(1-\phi_{m,t-1}\right)\right)\\ \text{and}\\ g_{m,t}{∼\text{Norm}|}_{0}^{+\infty }\left(n_{m,t-1}\times \gamma_{m,t-1},n_{m,t-1}\times \gamma_{m,t-1}\right) \end{array}$$

This parameterization provides a natural extension of IPMs for continuous data, whereby population dynamics are described by a shape parameter representing the previous year's population state and vital rate parameters representing net demographic losses and gains. The vital rate parameter in the survival mean model, $\phi_{m,t}$, is the adult apparent survival probability derived from a model of MAPS CMR data; and the $\gamma_{m,t}$ parameter in the recruitment mean model is a composite parameter that includes a mixture of fecundity, first‐year survival, and immigration components (models for vital rate parameters described in detail below). Interpretation of $\gamma_{m,t}$ should also be cautioned by acknowledgement that this parameter may also absorb unexplained variation representing discrepancies in the sampling process between data sets. We decomposed $\gamma_{m,t}$ into a productivity component derived from age‐specific MAPS data, RI*_m,t_*, and a latent parameter, *ι_m,t_*, which reflects variation in first‐year survival and immigration (i.e., local and external “recruits” of any age; DeSante, Kaschube, & Saracco, 2015), based on $\gamma_{m,t}={\text{RI}}_{m,t}\times \iota_{m,t}$.

We derived regional‐scale route‐level abundance indices as:

$$N_{m,t}=w_{m}\times \exp\left(\log\left(n_{m,t}\right)+0.5\times \sigma_{\omega_{i}}^{2}+0.5\times \sigma_{\varepsilon_{i,m,t}}^{2}\right)$$

where the *w_m_* are weights representing the proportion of BBS routes on which Wilson's warblers were encountered in the region and the $\sigma_{\omega_{i}}^{2}$ and $\sigma_{\varepsilon_{i,m,t}}^{2}$ are variance components of route × observer and overdispersion effects (Sauer & Link, 2011). We calculated regional population trends as geometric means of the annual realized population growth rates ($N_{m,t+1}/N_{m,t}$). For composite abundance and trend estimates, we weighted regional abundances by proportions of area encompassed by regions (Link & Sauer, 2002).

The stratum‐ and year‐specific adult survival probabilities, $\phi_{m,t}$ (Equation 1), were informed by individual encounter history data from the MAPS program modeled using a state‐space version of the Cormack‐Jolly Seber model that accounts for transients (i.e., individuals with zero probability of recapture after the year of marking; Pradel, Hines, Lebreton, & Nichols, 1997) in the data set (Saracco et al., 2010; dashed red box in Figure 3). The model assumes that the “alive state,” *z*, of individual *j* in stratum *m* and time *t* is a Bernoulli process with the probability parameter equal to the product of the individual's residency state, *R* (0 = transient; 1 = resident), its alive state in time *t* − 1 (0 = dead or permanently emigrated; 1 = alive and available for capture), and the apparent survival rate: $z_{j,m,t}|z_{j,m,t-1}∼\text{Bern}\left(R_{j,m,t-1}z_{j,m,t-1}\phi_{m,t-1}\right)$. We modeled residency state of newly marked individuals based on a Bernoulli distribution with residency probability parameter, $\pi_{m,t}$: $R_{j,m,t}∼\text{Bern}\left(\pi_{m,first(j)}\right)$, where first(*j*) indicates the year of marking for individual *j*.

We defined a logit‐linear model for $\phi_{m,t}$ that allowed survival to vary as a function of a stratum‐specific mean on logit scale ($\text{logit(}\phi_{0\left[m\right]})$), the winter drought index, cmd*_m,t_*, the tailwind covariate, tw*_m,t_*, and a zero‐mean random stratum‐specific year effect, $\nu_{m,t}$:

$$\text{logit}\left(\phi_{m,t}\right)=\text{logit}\left(\phi_{0[m]}\right)+\beta_{\text{cmd}\left[m\right]}\times {\text{cmd}}_{m,t}+\beta_{\text{tw}\left[m\right]}\times {\text{tw}}_{m,t}+\nu_{m,t}$$

We defined an analogous logit‐linear model for $\pi_{m,t}$ with the exception that we did not include the climate covariates. For the stratum‐specific year effects in both models, we allowed precision hyperparameters to be stratum‐specific.

Assuming independence among data sets, the likelihood of the IPM, *L*_IPM_, can be defined as the product of the likelihoods of the three‐component models, including the state‐space count model, *L*_SS_, the state‐space CJS model, *L*_CJS_, and the binomial model for the age‐specific capture data, *L*_Prod_ (Figure 3). The likelihood of the state‐space count model, *L*_SS_, can be defined as the product of likelihoods for the observation (*L*_O_) and system (*L*_S_) process models: $L_{\mathrm{SS}}\left(y|n,\omega ,\eta ,\varepsilon ,\phi ,RI,\iota \right)=L_{O}\left(y|n,\omega ,\eta ,\varepsilon \right)\times L_{S}\left(n|\phi ,RI,\iota \right)$; the likelihood for the CJS model (*L*_CJS_) can be defined as: $L_{\mathrm{CJS}}\left(c,r|z,R,\phi ,\pi ,mathbfp,\rho ,\beta_{\mathrm{cmd}},\beta_{\mathrm{tw}}\right)$; and the likelihood for the binomial productivity model can be defined as $L_{\mathrm{Pr}od}\left(\mathrm{HY}|\mathrm{Nind},\alpha_{0},\alpha_{\mathrm{ef}},\alpha_{\mathrm{cmd}},\alpha_{\mathrm{temp}},\mathrm{yr},\mathrm{sta}\right)$.

We implemented the model with JAGS 3.3.0 (Plummer, 2003) using the jags function of the jagsUI package (Kellner, 2015) in the R statistical computing environment (R Core Team, 2018). We assigned vague uniform U(0, 1) prior distributions for inverse‐logit transformed intercepts of models for parameters on 0–1 probability scales. Regression coefficients for fixed effects of linear models were modeled with Norm(0, 10^–3^) priors, and standard deviation hyperparameters were modeled with U(0, 10) priors. We inferred support for vital rate‐covariate relationships for regression coefficients with 95% credible intervals that did not overlap zero. The first‐year survival/immigration parameter, *ι_m,t_*, was determined based on a weakly informative prior distribution, ι∼Norm(1,100). This prior ensured only a plausible range of values for this parameter with prior mean consistent with results of previous MAPS analyses (DeSante et al., 2015). Posterior distributions of the demographic parameters and population size were derived from 80,000 simulated values of four chains from the posterior distribution after an adaptive phase of 40,000 iterations and burn‐in of 20,000 samples of the Gibbs sampler and thinning by 4. The Markov chains were determined to have successfully converged if $\hat{R}$ values were <1.1 for posterior estimates of all parameters (Gelman & Hill, 2006). We present all parameter estimates as means ± 95% credible intervals.

### 2.4 Demographic contributions to variation in population change

We used transient life table response experiments (LTREs) to decompose temporal variation in population growth rates among vital rate and demographic structure components (Koons et al., 2017, 2016). Specifically, we considered contributions of adult apparent survival, *ϕ*; productivity (of both new recruits/immigrants and survivors from previous time step), RI; first‐year survival/recruitment, *ι*. Following Koons et al. (2017), we also considered contributions of demographic age structure; however, as in that study, we found that age structure contributed virtually nothing to explaining variation in population growth. Thus, we do not report those results here. Finally, we examined annual changes in population growth rate in relation to changes in climate covariates to better understand how climate variation drives population dynamics.

## 3 RESULTS

### 3.1 Population status, trends, and vital rate dynamics

**Figure 4:**
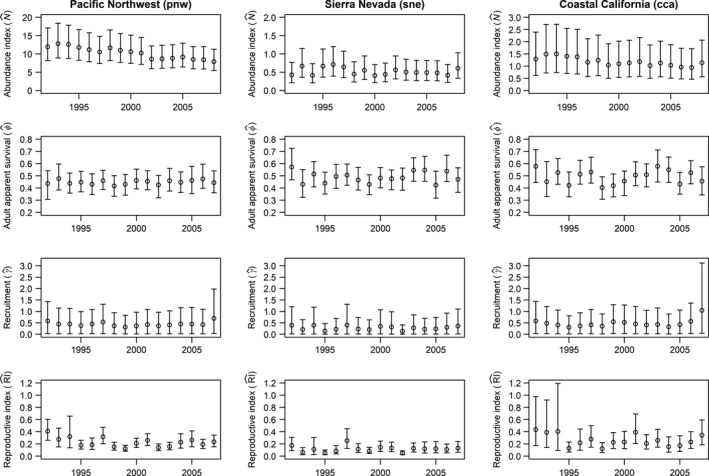
Annual estimates of abundance and demographic rates (means ± 95% credible intervals) for each Wilson's warbler population

Area‐weighted estimates of our population size index, suggest that the a pnw population [$\hat{N}$ = 10.26 (6.35, 15.88)] is about 9 × larger than the cca population [$\hat{N}$ = 1.19 (0.55, 2.27)], and 20 × larger than the sne [$\hat{N}$ = 0.53 (0.25, 0.98)] population. We estimate that the three Wilson's warbler genetic groups have declined overall by an average of 2.1%/year (−3.3%, −0.9%) over the 17‐year (1992–2007) study period [where trend = 100 × ($\hat{\lambda }$ − 1)]. This trend was largely driven by the relatively large pnw group, which declined by about 2.5%/year (−3.9%, −1.8%). The cca group declined less severely [−0.7%/year (−3.3%, +1.9%)], while the sne population showed evidence of positive population trend [+2.3%/year (−1.6%, +6.4%)]. Annual population growth rate estimates ($\hat{\lambda }$) were variable for the sne population (although precision was low) compared with the other two genetic groups (Figure 4).

Mean adult apparent survival rates were slightly higher for the California populations [0.50 (0.42, 0.58) for sne; 0.49 (0.42, 0.57) for cca] than for the Pacific Northwest [0.45 (0.41, 0.49)]. Adult survival was relatively stable and showed similar levels of annual variability across regions (mean ${\hat{\sigma }}_{\nu }$ ranging from 0.16 to 0.17; Figure 4). Point estimates of mean recruitment ($\hat{\gamma }$) were lower for the sne population [0.28 (0.01, 0.94)] than for the other two groups [0.48 (0.02, 1.40) for cca; 0.44 (0.02, 1.21) for pnw]; however, precision was low for all recruitment estimates (Figure 4). Mean productivity was lower for sne [0.10 (0.06, 0.17)] than for cca [0.23 (0.13, 0.39)] or pnw [0.21 (0.15, 0.29)]. Productivity was variable among years for all regions, albeit less so for pnw [${\hat{\sigma }}_{\text{yr}}$ = 0.25 (0.15, 0.41)] compared with the other two regions [${\hat{\sigma }}_{\text{yr}}$ = 0.40 (0.25, 0.63) for cca and ${\hat{\sigma }}_{\text{yr}}$ = 0.42 (0.26, 0.66) for sne].

### 3.2 Climate covariate relationships

**Figure 5:**
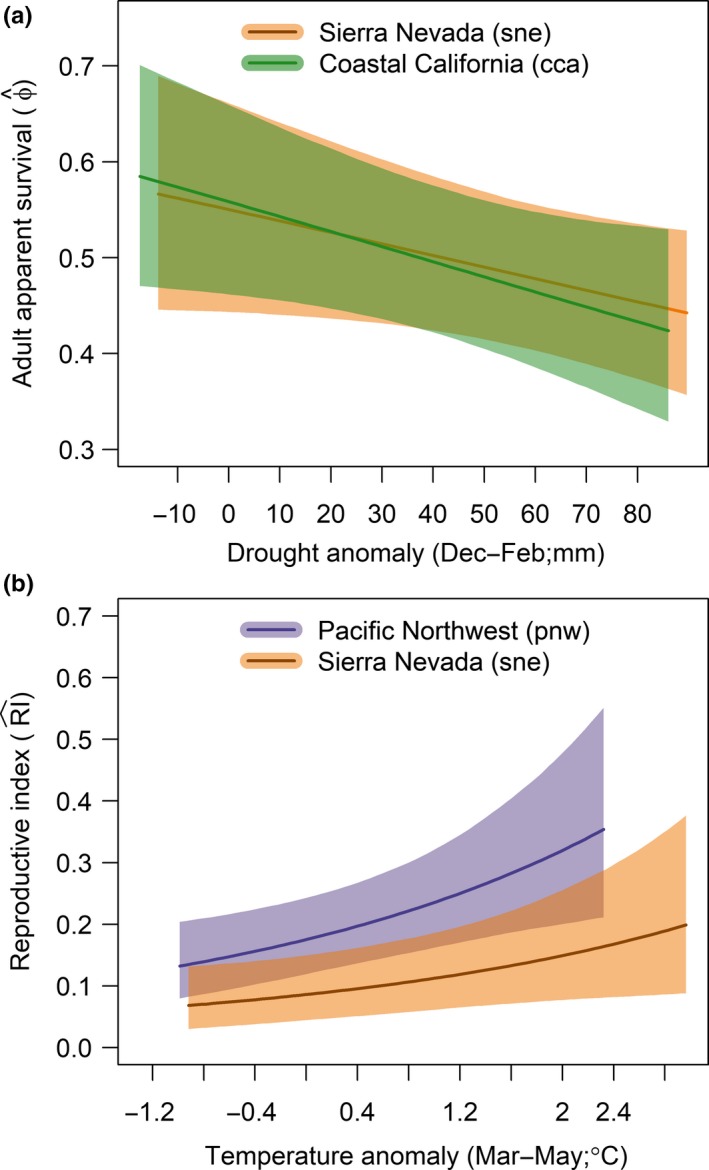
Estimated relationships between demographic parameters and climate covariates (means ± 95% credible intervals). (a) adult apparent survival probability declined with increasing drought anomaly for the Sierra Nevada (sne) and coastal California (cca) populations; and (b) productivity increased as a function of spring temperature anomaly for the sne and Pacific Northwest (pnw) populations

Adult apparent survival was negatively related to winter drought for the two California genetic groups for (Table 2, Figure 5a), but not for the pnw group (Table 2). We found no relationship between tailwind and adult apparent survival (Table 2). We found no evidence of a carry‐over effect of winter drought effect on productivity [${\hat{\alpha }}_{\text{cmd}}$ = 0.01 (−0.12, 0.13)]. Spring temperature was only weakly related to productivity for the cca group (Table 2); this relationship was much stronger for the sne and pnw groups (Table 2; Figure 5b).

**Table 2:** Mean (95% credible interval) coefficient estimates from logit‐linear models indicating vital rate‐covariate relationships for the three Wilson's warbler genetic groups

	Pacific Northwest (pne)	Sierra Nevada (sne)	Coastal California (cca)
Adult apparent survival (*ϕ*)			
Winter drought (${\hat{\beta }}_{\text{cmd}}$)	0.00 (−0.17, 0.19)	−0.17 (−0.38, 0.02)	−0.23 (−0.45, 0.00)
Tailwind (${\hat{\beta }}_{\text{tw}}$)	−0.06 (−0.38, 0.27)	0.03 (−0.23, 0.30)	0.04 (−0.19, 0.26)
Productivity (RI)			
Winter drought (${\hat{\alpha }}_{\text{cmd}}$)	−0.01 (−0.20, 0.16)	0.11 (−0.15, 0.36)	−0.08 (−0.38, 0.20)
Spring temperature (${\hat{\alpha }}_{\text{temp}}$)	0.28 (0.09, 0.48)	0.26 (0.03, 0.51)	0.06 (−0.20, 0.31)

### 3.3 Demographic and climatic contributions to population growth

**Figure 6:**
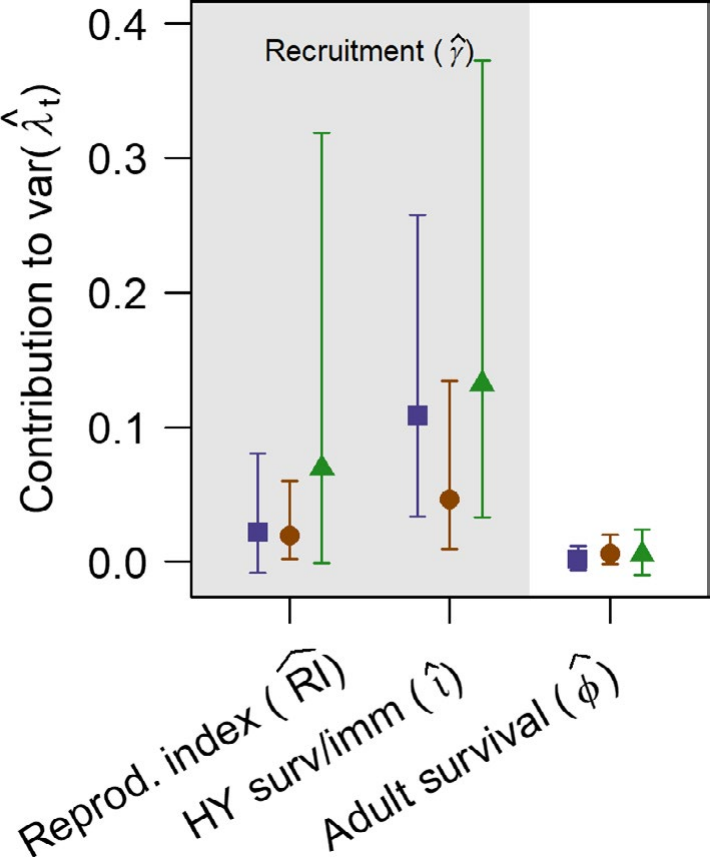
Demographic contributions to variation in population growth rate (means ± 95% credible intervals). The pnw population (left) is represented by blue squares, the sna population (middle) by orange circles, and the cca population (right) by green triangles. Recruitment parameters (RI and ι; gray region) contributed substantially more to explaining annual variation than did adult survival (ϕ; white region)

Annual changes in population growth rates were driven principally by recruitment components (RI and ι), rather than by changes in adult apparent survival (Figure 6). Our index of first‐year (HY) survival, ι, had the largest effect on annual population changes for all three populations.

**Figure 7:**
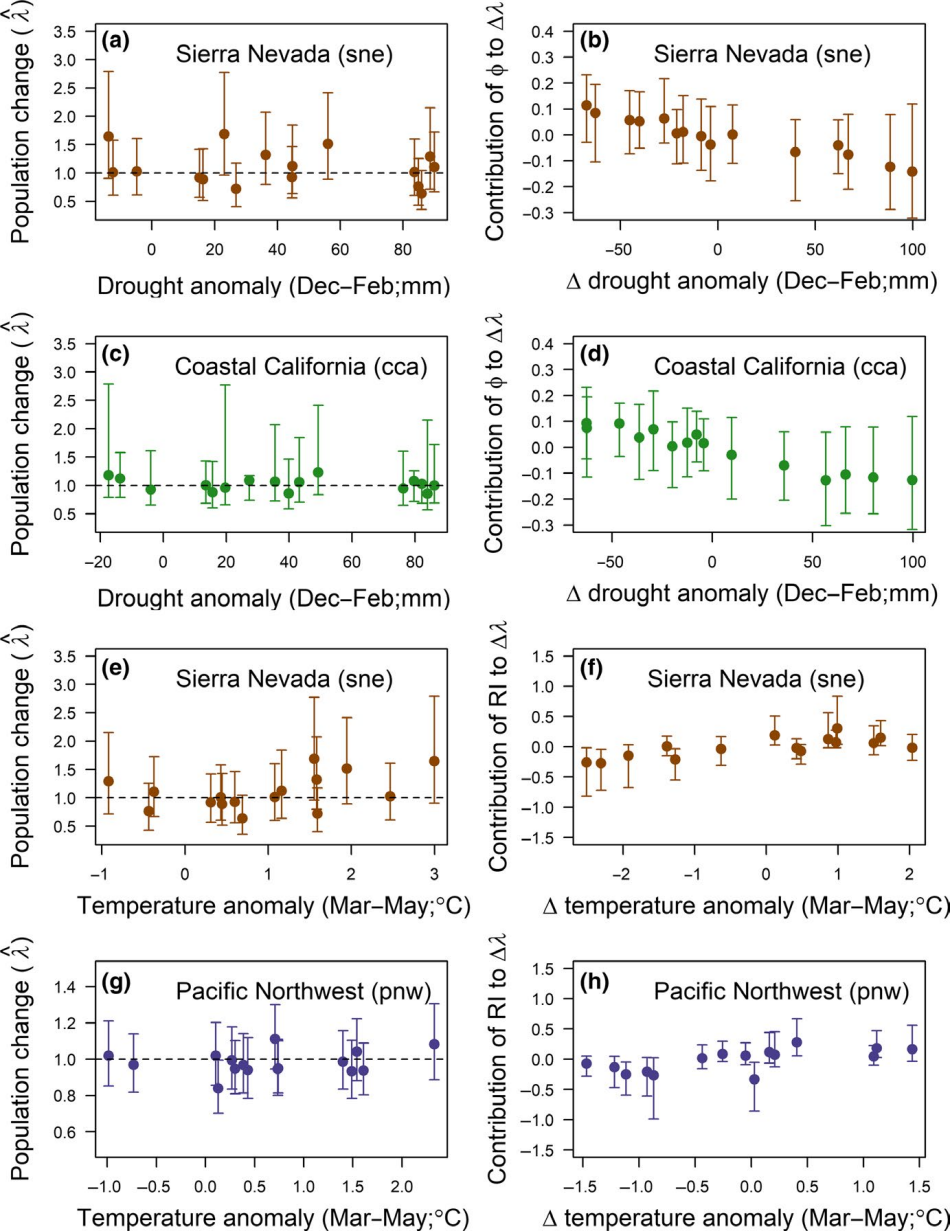
Annual population growth rates v. covariates that influenced vital rates (a, c, e) and contributions of demographic parameters to changes in population growth rates v. annual changes in climate covariate values (b, d, f). Points represent means and error bars delineate 95% credible intervals

We found relatively little evidence of a relationship between winter drought conditions and population change for the two California populations (Figure 7a,c). Nevertheless, differences in drought conditions between years did appear to be related to differences in population growth between years (Figure 7b,d). For example, transitions between severe drought conditions in winter 1993–1994 to more normal conditions in winter 1994–1995 corresponded to a noticeable increase in survival between the 1993 and 1994 survival intervals for the cca and sne regions and a significant population increase in the sne region during that interval (Figure 4). Return to drought conditions the following year was marked by survival declines in all three genetic groups.

Contributions of annual changes in spring temperature to annual changes in population growth rate were relatively large for the sne (Figure 7e,f) and pnw (Figure 7g,h) populations. Declines in spring temperature between years of ~<1°C tended to be associated with negative contributions to changes in population growth, while positive changes in population growth (at least up to ~1°C) tended to be associated with positive contributions to changes in population growth (Figure 7f,h).

## 4 DISCUSSION

Integrated population models have gained wide usage in population ecology because they provide a cohesive framework for understanding demographic and environmental drivers of population change (Koons et al., 2017; Schaub & Abadi, 2011). However, these models have received little attention in applications that combine multiple independent surveys across broad spatial and temporal scales (Ahrestani et al., 2017; Robinson et al., 2014; Zhao, Boomer, & Kendall, 2018). The IPM presented here provides a flexible framework for these broad‐scale multi‐site applications by modeling the survival and recruitment processes as functions of continuous random variables, rather than as functions of binomial and poisson processes typical of most IPM applications. We follow Brintz, Fuentes, and Madsen (2018) and Zhao et al. (2019) by modeling the demographic processes based on a Gaussian approximations of binomial and Poisson models. Another option that we have found yielded similar results was to use gamma distributions with shape parameters determined by population state and rate parameters determined by the demographic rates. These approaches for continuous data provide a natural means of linking count and CMR data collected at different collections of sites at common regional scales. Use of discrete models in these situations (Ahrestani et al., 2017) can be problematic when average population counts are low for a region (e.g., at the periphery of the range as in our cca and sne regions) and susceptible to extinction. In this discrete model scenario, a separate immigration parameter would be needed to allow for regional recovery (Abadi, Gimenez, Ullrich, Arlettaz, & Schaub, 2010; Hostetler & Chandler, 2015; Schaub & Fletcher, 2015; Schaub, Jakober, & Stauber, 2013). However, as in our application, scales of local and external recruitment are not always clear, and introducing this parameter creates additional latency that can complicate or preclude reliable estimation. Given already low precision of estimates for many of the fully time‐specific parameters in our model, particularly for the sne and cca populations for which we had relatively few data, it would be difficult to justify this additional complication.

Despite low precision of recruitment estimates, our results suggested that greater variation in productivity and recruitment than in adult survival, and that recruitment played a much larger role in explaining annual variation in population change than did adult survival. These findings are likely due, in part, to discrepancies between MAPS and BBS data sets, which differ in sizes and spatio‐temporal distribution of sampling units. Thus, ecological interpretation of the recruitment component of our model must be tempered due to inclusion of error associated with discrepancies in the sampling processes between data sets (Riecke, Leach, Gibson, & Sedinger, 2018). Alternative models for the capture‐recapture data, such as Jolly Seber (JS) models (Link & Barker, 2005; Royle & Dorazio, 2008) could be incorporated to inform recruitment directly. However, we have found these models impractical with large data sets due to long computation times, especially whenever capture probabilities are low (as in our example) because of the necessary addition of large numbers of additional all‐zero capture histories. Reverse‐symmetry models based on site‐level data summaries could also be used (Pradel, 1996; Tenan et al., 2014); however, we have encountered long implementation times with these models and difficulty achieving convergence when there are large numbers of missing site × year combinations. Furthermore, both of these solutions require modification to account for transiency, which is not straightforward in the context of JS or reverse‐symmetry models. Despite having little direct information on recruitment in our model and imprecise estimates of the first‐year survival/immigration component of recruitment (ι), our results implicating the importance of recruitment in explaining temporal variation in population growth rates are consistent with results of analyses based on MAPS data alone for a variety of bird species, including Wilson's warbler (DeSante et al., 2015; Wilson et al., 2018).

We linked demographic parameters to climate covariates that will be impacted by climate change. Spring winds have been shown to affect survival in other passerine birds (Drake et al., 2014; Huang et al., 2017); however, we found no evidence of spring wind effects on these three Wilson's warbler groups. It is possible that our wind covariate did not properly capture migration conditions due to how migration regions were delineated or that mean tailwind conditions across the entire migration period was not an appropriate metric. However, similar measures, such as number of days with positive tailwind, were strongly correlated with mean tailwind, suggesting that, if wind were an important predictor of survival, we might have detected it. It should be noted, however, that across our study regions, spring tailwinds tend to be negative (i.e., they are headwinds), particularly along the coast. Because of increased coastal upwelling, these winds are predicted to become more negative (i.e., stronger northerly) under climate change (Sydeman et al., 2014), which will likely exacerbate the potential for a negative effects of headwinds on survival in the coming decades. We did find significant negative effects of winter drought conditions on adult apparent survival for the California populations. It is possible that drought conditions typical of late summers in California (prior to fall migration) could leave these populations more vulnerable to drought on their wintering grounds than populations of the Pacific Northwest that do not normally experience pronounced late summer drought. Average drought conditions are expected to worsen in western Mexico under climate change scenarios, where these genetic groups of Wilson's warbler overwinter (Trenberth et al., 2013). Although our results suggested that adult apparent survival contributed less to overall population trends than recruitment, conserving and managing for drought‐resilience habitats will nevertheless likely be an important component of any conservation plan for these populations.

Our analysis incorporated age‐specific capture data to model postfledging productivity, which allows assessment of relative contributions of reproductive output and first‐year survival and immigration to recruitment and population change. Although we found no evidence of winter drought carry‐over effects on productivity, we did find population‐specific effects of spring temperature on productivity: Productivity of populations in the montane (sne) and northerly (pnw) regions increased with increasing spring temperature. This finding is consistent with results of multi‐species productivity models applied to MAPS data within the sne region (Saracco et al., 2019). Productivity was an important contributor to annual variation in population change. For the sne and pnw populations, negative changes in spring temperatures between years tended to result in negative contributions to between‐year changes in population growth, while years with increases in temperature over the previous year tended to yield positive contributions of productivity to changes in population growth. It should be noted, however, that there was some indication that positive temperature effects declined at larger between‐year spring temperature increases (~>1°C increases), which may have implications under an increasingly variable and warming environment.

Large‐scale multi‐site surveys have long played an important role in advancing ecology and conservation (Buckland, Magurran, Green, & Fewster, 2005; Magurran et al., 2016). Continued development of IPMs that combine large‐scale data sets of marked individuals with structured (e.g., BBS) or unstructured (e.g., eBird; Robinson et al., 2018) observational data should lend powerful new insights into the status and trends of populations as they encounter novel environments associated with recent habitat and climate change (Butchart et al., 2010; Tittensor et al., 2014). Although we do not explore predictions of future populations here, by extending the time series of our model into the future, extinction risk for each of the genetic groups could be assessed based on predicted time series of future climate covariate values and estimates of mean demographic rates and demographic stochasticity (our hierarchical region‐specific estimates of annual variation). Such analyses could assist in weighing the potential effectiveness of various conservation actions in the context of environmental trends and variation (Boyce, Haridas, Lee, & The NCEAS Stochastic Demography Working Group, 2006; Lawson, Vindenes, Bailey, & van de Pol, 2015) and alternative conservation priorities (e.g., conservation of rare genetic lineages v. conservation of the most individuals; Ruegg et al., unpublished data).

## CONFLICT OF INTEREST

None declared.

## AUTHOR CONTRIBUTIONS

Both authors conceived the study. JS prepared the data, developed the model, analyzed the data, and led the writing of the manuscript. Both authors contributed critically to drafts and approved the final version for publication.

### Open Research Badges

This article has earned an Open Data Badge for making publicly available the digitally‐shareable data necessary to reproduce the reported results. The data is available at https://doi.org/10.21429/04ma-p963.

## Data Availability

Data and code for implementing the model and reproducing results have been deposited in USGS's Sciencebase (https://www.sciencebase.gov/catalog/): https://doi.org/10.21429/04ma-p963.
